# Supplementary material for: Multi‐dataset identification of innovative feature genes and molecular mechanisms in keratoconus
Source: J Cell Mol Med. 2024 Sep 19;28(18):e70079. doi: 10.1111/jcmm.70079 (PMC11412914; doi:10.1111/jcmm.70079)
Supplement: Supplementary file 2 — Material S2. [file JCMM-28-e70079-s002.zip › 7.GSEA analysis/6.SNORA74B/KEGG_PANTOTHENATE_AND_COA_BIOSYNTHESIS.html]

Details for gene set KEGG\_PANTOTHENATE\_AND\_COA\_BIOSYNTHESIS[GSEA]

|  || Dataset | SNORA74B.group.cls#h\_versus\_l |
| Phenotype | group.cls#h\_versus\_l |
| Upregulated in class | l |
| GeneSet | KEGG\_PANTOTHENATE\_AND\_COA\_BIOSYNTHESIS |
| Enrichment Score (ES) | -0.52439195 |
| Normalized Enrichment Score (NES) | -1.3660032 |
| Nominal p-value | 0.13948919 |
| FDR q-value | 0.3366099 |
| FWER p-Value | 0.92 |
Table: GSEA Results Summary

  

Fig 1: Enrichment plot: KEGG\_PANTOTHENATE\_AND\_COA\_BIOSYNTHESIS      
 Profile of the Running ES Score & Positions of GeneSet Members on the Rank Ordered List

  

| SYMBOL | TITLE | RANK IN GENE LIST | RANK METRIC SCORE | RUNNING ES | CORE ENRICHMENT || 1 | PPCS | na | 10473 | 0.084 | -0.2468 | No |
| 2 | DPYS | na | 10845 | 0.077 | -0.2066 | No |
| 3 | UPB1 | na | 11630 | 0.065 | -0.1865 | No |
| 4 | ENPP3 | na | 12163 | 0.058 | -0.1640 | No |
| 5 | BCAT1 | na | 14571 | 0.029 | -0.2141 | No |
| 6 | PANK2 | na | 14764 | 0.027 | -0.2020 | No |
| 7 | PANK3 | na | 15305 | 0.021 | -0.2034 | No |
| 8 | PANK1 | na | 16367 | 0.011 | -0.2269 | No |
| 9 | BCAT2 | na | 17817 | -0.004 | -0.2661 | No |
| 10 | COASY | na | 17959 | -0.006 | -0.2665 | No |
| 11 | VNN1 | na | 18436 | -0.011 | -0.2729 | No |
| 12 | VNN2 | na | 27166 | -0.143 | -0.4305 | Yes |
| 13 | PPCDC | na | 28354 | -0.175 | -0.3498 | Yes |
| 14 | PANK4 | na | 28516 | -0.180 | -0.2363 | Yes |
| 15 | DPYD | na | 28535 | -0.180 | -0.1184 | Yes |
| 16 | ENPP1 | na | 33524 | -0.452 | 0.0347 | Yes |
Table: GSEA details [plain text format]

  

Fig 2: KEGG\_PANTOTHENATE\_AND\_COA\_BIOSYNTHESIS      
 Blue-Pink O' Gram in the Space of the Analyzed GeneSet

  

Fig 3: KEGG\_PANTOTHENATE\_AND\_COA\_BIOSYNTHESIS: Random ES distribution      
 Gene set null distribution of ES for **KEGG\_PANTOTHENATE\_AND\_COA\_BIOSYNTHESIS**
